# 5-Methyl 3-(2-methyl­prop-3-yl) 2,6-di­methyl-4-(2-nitro­sophen­yl)pyridine-3,5-dicarboxyl­ate

**DOI:** 10.1107/S1600536810005088

**Published:** 2010-02-13

**Authors:** Hui Chen, Ding Qu, Qiao-Feng Wang, Ru Jiang

**Affiliations:** a School of Pharmacy, Fourth Military Medical University, Changle West Road 17, 710032 Xi-An, People’s Republic of China

## Abstract

In the title compound, C_20_H_22_N_2_O_5_, a photo-degradation product of the hypertension drug nisoldipine, the dihedral angle between the nitro­sophenyl ring and the pyridine ring is 75.7 (3)°. In the crystal structure, weak C—H⋯O hydrogen bonds help to establish the packing.

## Related literature

For general background to nisoldipine derivatives, see: Marciniec *et al.* (2002 ▶).
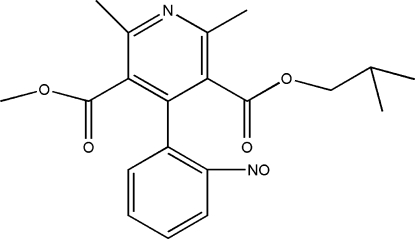


## Experimental

### 

#### Crystal data

C_20_H_22_N_2_O_5_
*M* *_r_* = 370.40
Triclinic, 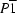

*a* = 7.1831 (4) Å
*b* = 9.7819 (6) Å
*c* = 15.0245 (9) Å
α = 89.488 (3)°
β = 81.201 (3)°
γ = 70.625 (3)°
*V* = 983.19 (10) Å^3^
*Z* = 2
Mo *K*α radiation
μ = 0.09 mm^−1^
*T* = 296 K
0.42 × 0.28 × 0.22 mm

#### Data collection

Bruker APEX II CCD diffractometer
Absorption correction: multi-scan (*SADABS*; Bruker, 2001 ▶) *T*_min_ = 0.963, *T*_max_ = 0.980
5353 measured reflections
3623 independent reflections
2500 reflections with *I* > 2σ(*I*)
*R*_int_ = 0.015

#### Refinement

*R*[*F* ^2^ > 2σ(*F* ^2^)] = 0.077
*wR*(*F* ^2^) = 0.311
*S* = 1.01
3623 reflections
250 parameters
H-atom parameters not refined
Δρ_max_ = 0.81 e Å^−3^
Δρ_min_ = −0.29 e Å^−3^

### 

Data collection: *APEX2* (Bruker, 2004 ▶); cell refinement: *SAINT-Plus* (Bruker, 2001 ▶); data reduction: *SAINT-Plus*; program(s) used to solve structure: *SHELXS97* (Sheldrick, 2008 ▶); program(s) used to refine structure: *SHELXL97* (Sheldrick, 2008 ▶); molecular graphics: *SHELXTL* (Sheldrick, 2008 ▶); software used to prepare material for publication: *SHELXTL*.

## Supplementary Material

Crystal structure: contains datablocks global, I. DOI: 10.1107/S1600536810005088/hb5320sup1.cif

Structure factors: contains datablocks I. DOI: 10.1107/S1600536810005088/hb5320Isup2.hkl

Additional supplementary materials:  crystallographic information; 3D view; checkCIF report

## Figures and Tables

**Table 1:** Hydrogen-bond geometry (Å, °)

*D*—H⋯*A*	*D*—H	H⋯*A*	*D*⋯*A*	*D*—H⋯*A*
C13—H13*B*⋯O1^i^	0.96	2.39	3.344 (5)	172
C14—H14*B*⋯O4^ii^	0.96	2.52	3.472 (5)	174

Symmetry codes: (i) 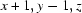; (ii) 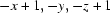.

**Fig. 1.**
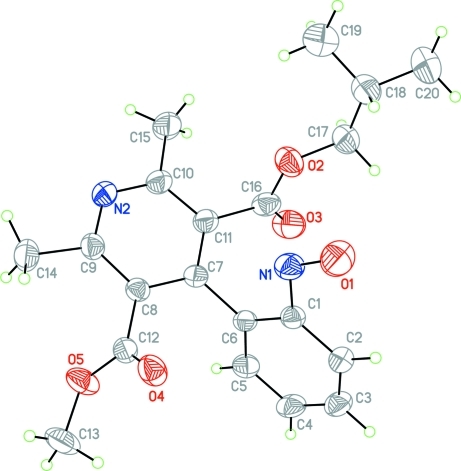
The molecular structure of (I) with displacement ellipsoids drawn at the 30% probability level; H atoms are given as spheres of arbitrary radius.

## supplementary crystallographic information

### Comment

Nisoldipine has been the subject of many analytical chemical investigations due to the commercial preparations for treatment of hypertension (Marciniec *et al.*, 2002). Here, we describe the synthesis and structural characterization of the title compound.

The molecular structure of the title compound is shown in Fig. 1. In this structure, the dihedral angle between the nitrosophenyl ring and the pyridine ring is 75.7 (3)°. Weak C—H···O hydrogen bonding between the cations and anions leads to a consolidation of the structure.

### Experimental

A solution of nisoldipine (10 mmol) in 50 ml acetone was exposed to sunlight for 5 h at ambient temperature. To the mixture was added 50 ml water, followed by filtration. The crude product was purified by flash chromatography on silica gel (1:1 ethyl acetate/hexane). Anal. C_20_H_22_N_2_O_5_: C, 64.79; H, 5.94; N, 7.56. Found: C, 64.65; H, 5.82; N, 7.50 %. Colourless blocks of (I) were recrystallised from ethanol.

### Refinement

H-atoms were included in calculated positions and treated as riding atoms: C—H = 0.92—0.96 Å with Uiso(H) = 1.2Ueq(C) or 1.5Ueq(CMe). In the final difference map the highest peak is 1.68 Å from atom O3 and the deepest hole is 0.65 Å from atom O2.

### Crystal data

**Table:**

C_20_H_22_N_2_O_5_	*Z* = 2
*M**_r_* = 370.40	*F*(000) = 392
Triclinic, *P*1	*D*_x_ = 1.251 Mg m^−^^3^
Hall symbol: -P 1	Mo *K*α radiation, λ = 0.71073 Å
*a* = 7.1831 (4) Å	Cell parameters from 1617 reflections
*b* = 9.7819 (6) Å	θ = 2.2–24.3°
*c* = 15.0245 (9) Å	µ = 0.09 mm^−^^1^
α = 89.488 (3)°	*T* = 296 K
β = 81.201 (3)°	Block, colourless
γ = 70.625 (3)°	0.42 × 0.28 × 0.22 mm
*V* = 983.19 (10) Å^3^	

### Data collection

**Table:**

Bruker APEX II CCD diffractometer	3623 independent reflections
Radiation source: fine-focus sealed tube	2500 reflections with *I* > 2σ(*I*)
graphite	*R*_int_ = 0.015
φ and ω scans	θ_max_ = 25.5°, θ_min_ = 2.2°
Absorption correction: multi-scan (*SADABS*; Bruker, 2001)	*h* = −8→8
*T*_min_ = 0.963, *T*_max_ = 0.980	*k* = −11→11
5353 measured reflections	*l* = −15→18

### Refinement

**Table:**

Refinement on *F*^2^	Primary atom site location: structure-invariant direct methods
Least-squares matrix: full	Secondary atom site location: difference Fourier map
*R*[*F*^2^ > 2σ(*F*^2^)] = 0.077	Hydrogen site location: inferred from neighbouring sites
*wR*(*F*^2^) = 0.311	H-atom parameters not refined
*S* = 1.01	*w* = 1/[σ^2^(*F*_o_^2^) + (0.240*P*)^2^] where *P* = (*F*_o_^2^ + 2*F*_c_^2^)/3
3623 reflections	(Δ/σ)_max_ < 0.001
250 parameters	Δρ_max_ = 0.81 e Å^−^^3^
0 restraints	Δρ_min_ = −0.28 e Å^−^^3^

### Special details

**Table:**

Geometry. All esds (except the esd in the dihedral angle between two l.s. planes) are estimated using the full covariance matrix. The cell esds are taken into account individually in the estimation of esds in distances, angles and torsion angles; correlations between esds in cell parameters are only used when they are defined by crystal symmetry. An approximate (isotropic) treatment of cell esds is used for estimating esds involving l.s. planes.
Refinement. Refinement of *F*^2^ against ALL reflections. The weighted *R*-factor wR and goodness of fit *S* are based on *F*^2^, conventional *R*-factors *R* are based on *F*, with *F* set to zero for negative *F*^2^. The threshold expression of *F*^2^ > σ(*F*^2^) is used only for calculating *R*-factors(gt) etc. and is not relevant to the choice of reflections for refinement. *R*-factors based on *F*^2^ are statistically about twice as large as those based on *F*, and *R*- factors based on ALL data will be even larger.

### Fractional atomic coordinates and isotropic or equivalent isotropic displacement parameters (Å^2^)

**Table:**

	*x*	*y*	*z*	*U*_iso_*/*U*_eq_	
C1	0.6145 (4)	0.4193 (3)	0.3529 (2)	0.0509 (7)	
C2	0.7137 (5)	0.5026 (4)	0.3888 (2)	0.0619 (8)	
H2	0.6424	0.5893	0.4215	0.074*	
C3	0.9183 (5)	0.4525 (4)	0.3742 (2)	0.0686 (10)	
H3	0.9872	0.5068	0.3963	0.082*	
C4	1.0230 (5)	0.3236 (4)	0.3276 (2)	0.0655 (9)	
H4	1.1620	0.2908	0.3190	0.079*	
C5	0.9240 (4)	0.2413 (4)	0.2931 (2)	0.0597 (8)	
H5	0.9969	0.1538	0.2615	0.072*	
C6	0.7173 (4)	0.2887 (3)	0.30541 (18)	0.0471 (7)	
C7	0.6111 (4)	0.2014 (3)	0.26614 (18)	0.0486 (7)	
C8	0.5973 (4)	0.0750 (3)	0.30544 (19)	0.0493 (7)	
C9	0.5000 (5)	−0.0045 (3)	0.2660 (2)	0.0573 (8)	
C10	0.4346 (5)	0.1561 (4)	0.1519 (2)	0.0624 (8)	
C11	0.5245 (5)	0.2442 (3)	0.1897 (2)	0.0589 (8)	
C12	0.6737 (4)	0.0337 (3)	0.3918 (2)	0.0527 (7)	
C13	0.8802 (7)	−0.1539 (4)	0.4692 (3)	0.0913 (13)	
H13A	0.8912	−0.0762	0.5043	0.137*	
H13B	1.0089	−0.2278	0.4550	0.137*	
H13C	0.7878	−0.1942	0.5030	0.137*	
C14	0.4632 (7)	−0.1378 (4)	0.3078 (3)	0.0812 (11)	
H14A	0.3521	−0.1524	0.2859	0.122*	
H14B	0.4338	−0.1233	0.3722	0.122*	
H14C	0.5803	−0.2214	0.2915	0.122*	
C15	0.3426 (7)	0.1944 (5)	0.0689 (3)	0.0908 (13)	
H15A	0.3202	0.1112	0.0453	0.136*	
H15B	0.4307	0.2246	0.0246	0.136*	
H15C	0.2174	0.2720	0.0831	0.136*	
C16	0.5224 (5)	0.3875 (4)	0.1491 (2)	0.0652 (9)	
C17	0.3105 (6)	0.6240 (4)	0.1237 (3)	0.0798 (11)	
H17A	0.3503	0.6115	0.0589	0.096*	
H17B	0.3922	0.6714	0.1479	0.096*	
C18	0.0950 (7)	0.7135 (4)	0.1464 (3)	0.0862 (12)	
H18	0.0635	0.7300	0.2120	0.103*	
C19	−0.0422 (8)	0.6433 (6)	0.1188 (4)	0.1166 (18)	
H19A	−0.0070	0.6179	0.0554	0.175*	
H19B	−0.1771	0.7092	0.1313	0.175*	
H19C	−0.0314	0.5573	0.1519	0.175*	
C20	0.0630 (9)	0.8621 (5)	0.1047 (4)	0.1198 (18)	
H20A	0.1012	0.8489	0.0404	0.180*	
H20B	0.1431	0.9096	0.1287	0.180*	
H20C	−0.0755	0.9207	0.1188	0.180*	
N1	0.4005 (4)	0.4598 (3)	0.36627 (18)	0.0646 (8)	
N2	0.4221 (4)	0.0355 (3)	0.19043 (18)	0.0635 (7)	
O1	0.3121 (4)	0.5800 (3)	0.4003 (2)	0.0954 (10)	
O2	0.3373 (4)	0.4833 (3)	0.16329 (17)	0.0769 (8)	
O3	0.6613 (4)	0.4107 (3)	0.1127 (2)	0.0913 (9)	
O4	0.6244 (4)	0.1108 (2)	0.45823 (15)	0.0714 (7)	
O5	0.8089 (4)	−0.0987 (2)	0.38649 (16)	0.0771 (8)	

### Atomic displacement parameters (Å^2^)

**Table:**

	*U*^11^	*U*^22^	*U*^33^	*U*^12^	*U*^13^	*U*^23^
C1	0.0461 (15)	0.0539 (16)	0.0569 (16)	−0.0199 (13)	−0.0133 (12)	0.0081 (13)
C2	0.069 (2)	0.0572 (18)	0.0674 (19)	−0.0264 (15)	−0.0207 (16)	0.0038 (14)
C3	0.067 (2)	0.082 (2)	0.076 (2)	−0.0430 (18)	−0.0258 (17)	0.0156 (18)
C4	0.0428 (16)	0.083 (2)	0.078 (2)	−0.0264 (16)	−0.0173 (15)	0.0088 (18)
C5	0.0468 (16)	0.0646 (19)	0.0653 (18)	−0.0144 (14)	−0.0108 (14)	0.0018 (14)
C6	0.0467 (15)	0.0532 (16)	0.0468 (14)	−0.0213 (12)	−0.0142 (12)	0.0114 (12)
C7	0.0434 (14)	0.0519 (16)	0.0500 (15)	−0.0143 (12)	−0.0095 (12)	0.0033 (12)
C8	0.0463 (15)	0.0454 (15)	0.0526 (15)	−0.0094 (12)	−0.0104 (12)	0.0016 (12)
C9	0.0680 (19)	0.0475 (16)	0.0566 (17)	−0.0188 (14)	−0.0123 (14)	0.0018 (13)
C10	0.069 (2)	0.067 (2)	0.0594 (18)	−0.0290 (16)	−0.0223 (15)	0.0063 (15)
C11	0.0603 (18)	0.0646 (19)	0.0587 (17)	−0.0262 (15)	−0.0180 (14)	0.0102 (14)
C12	0.0525 (16)	0.0487 (16)	0.0580 (17)	−0.0183 (13)	−0.0093 (13)	0.0062 (13)
C13	0.102 (3)	0.075 (2)	0.086 (3)	−0.001 (2)	−0.046 (2)	0.018 (2)
C14	0.118 (3)	0.066 (2)	0.078 (2)	−0.048 (2)	−0.032 (2)	0.0136 (17)
C15	0.135 (4)	0.091 (3)	0.074 (2)	−0.057 (3)	−0.056 (2)	0.020 (2)
C16	0.069 (2)	0.084 (2)	0.0529 (17)	−0.0330 (19)	−0.0217 (16)	0.0143 (16)
C17	0.096 (3)	0.069 (2)	0.081 (2)	−0.030 (2)	−0.027 (2)	0.0173 (18)
C18	0.094 (3)	0.067 (2)	0.093 (3)	−0.015 (2)	−0.030 (2)	0.0032 (19)
C19	0.104 (4)	0.104 (4)	0.146 (5)	−0.031 (3)	−0.043 (3)	0.018 (3)
C20	0.139 (5)	0.073 (3)	0.144 (5)	−0.022 (3)	−0.041 (4)	0.015 (3)
N1	0.0495 (15)	0.0658 (17)	0.0748 (17)	−0.0131 (12)	−0.0125 (13)	−0.0023 (14)
N2	0.0762 (18)	0.0596 (16)	0.0632 (16)	−0.0274 (13)	−0.0253 (13)	0.0026 (12)
O1	0.0593 (15)	0.0809 (18)	0.132 (2)	−0.0068 (13)	−0.0088 (16)	−0.0327 (17)
O2	0.0765 (17)	0.0701 (15)	0.0836 (17)	−0.0239 (13)	−0.0133 (13)	0.0226 (12)
O3	0.0819 (18)	0.114 (2)	0.0919 (19)	−0.0493 (16)	−0.0209 (15)	0.0420 (17)
O4	0.0996 (19)	0.0565 (14)	0.0532 (13)	−0.0172 (12)	−0.0177 (12)	0.0036 (10)
O5	0.0776 (16)	0.0629 (14)	0.0695 (14)	0.0105 (12)	−0.0246 (12)	0.0006 (11)

### Geometric parameters (Å, °)

**Table:**

C1—C2	1.405 (4)	C13—H13A	0.9600
C1—N1	1.436 (4)	C13—H13B	0.9600
C1—C6	1.384 (4)	C13—H13C	0.9600
C2—C3	1.369 (5)	C14—H14A	0.9600
C2—H2	0.9300	C14—H14B	0.9600
C3—C4	1.368 (5)	C14—H14C	0.9600
C3—H3	0.9300	C15—H15A	0.9601
C4—C5	1.387 (5)	C15—H15B	0.9601
C4—H4	0.9300	C15—H15C	0.9601
C5—C6	1.383 (4)	C16—O3	1.152 (4)
C5—H5	0.9300	C16—O2	1.335 (4)
C6—C7	1.496 (4)	C17—O2	1.458 (4)
C7—C11	1.386 (4)	C17—C18	1.491 (6)
C7—C8	1.392 (4)	C17—H17A	0.9700
C8—C9	1.393 (4)	C17—H17B	0.9700
C8—C12	1.487 (4)	C18—C19	1.481 (7)
C9—N2	1.340 (4)	C18—C20	1.538 (6)
C9—C14	1.525 (4)	C18—H18	0.9800
C10—N2	1.333 (4)	C19—H19A	0.9600
C10—C11	1.404 (4)	C19—H19B	0.9600
C10—C15	1.487 (4)	C19—H19C	0.9600
C11—C16	1.520 (4)	C20—H20A	0.9600
C12—O4	1.191 (4)	C20—H20B	0.9600
C12—O5	1.331 (4)	C20—H20C	0.9600
C13—O5	1.448 (4)	N1—O1	1.210 (4)
			
C2—C1—N1	122.6 (3)	C9—C14—H14B	109.5
C2—C1—C6	122.0 (3)	H14A—C14—H14B	109.5
N1—C1—C6	115.3 (2)	C9—C14—H14C	109.5
C3—C2—C1	118.0 (3)	H14A—C14—H14C	109.5
C3—C2—H2	121.0	H14B—C14—H14C	109.5
C1—C2—H2	121.0	C10—C15—H15A	109.5
C2—C3—C4	120.9 (3)	C10—C15—H15B	109.5
C2—C3—H3	119.6	H15A—C15—H15B	109.5
C4—C3—H3	119.5	C10—C15—H15C	109.4
C3—C4—C5	120.7 (3)	H15A—C15—H15C	109.5
C3—C4—H4	119.8	H15B—C15—H15C	109.5
C5—C4—H4	119.6	O3—C16—O2	125.0 (3)
C6—C5—C4	120.3 (3)	O3—C16—C11	124.8 (3)
C6—C5—H5	119.9	O2—C16—C11	110.2 (3)
C4—C5—H5	119.8	O2—C17—C18	107.9 (3)
C5—C6—C1	118.1 (3)	O2—C17—H17A	110.2
C5—C6—C7	120.1 (3)	C18—C17—H17A	110.2
C1—C6—C7	121.8 (2)	O2—C17—H17B	110.1
C11—C7—C8	118.4 (3)	C18—C17—H17B	110.0
C11—C7—C6	120.8 (3)	H17A—C17—H17B	108.5
C8—C7—C6	120.8 (2)	C19—C18—C17	113.8 (4)
C9—C8—C7	119.0 (3)	C19—C18—C20	111.3 (4)
C9—C8—C12	121.5 (3)	C17—C18—C20	108.8 (4)
C7—C8—C12	119.3 (2)	C19—C18—H18	107.5
N2—C9—C8	122.1 (3)	C17—C18—H18	107.6
N2—C9—C14	114.9 (3)	C20—C18—H18	107.6
C8—C9—C14	122.9 (3)	C18—C19—H19A	109.4
N2—C10—C11	121.4 (3)	C18—C19—H19B	109.5
N2—C10—C15	116.5 (3)	H19A—C19—H19B	109.5
C11—C10—C15	122.1 (3)	C18—C19—H19C	109.6
C7—C11—C10	119.4 (3)	H19A—C19—H19C	109.5
C7—C11—C16	119.9 (3)	H19B—C19—H19C	109.5
C10—C11—C16	120.7 (3)	C18—C20—H20A	109.4
O4—C12—O5	123.1 (3)	C18—C20—H20B	109.3
O4—C12—C8	124.3 (3)	H20A—C20—H20B	109.5
O5—C12—C8	112.5 (2)	C18—C20—H20C	109.7
O5—C13—H13A	109.5	H20A—C20—H20C	109.5
O5—C13—H13B	109.5	H20B—C20—H20C	109.5
H13A—C13—H13B	109.5	O1—N1—C1	114.7 (3)
O5—C13—H13C	109.4	C10—N2—C9	119.6 (3)
H13A—C13—H13C	109.5	C16—O2—C17	116.4 (3)
H13B—C13—H13C	109.5	C12—O5—C13	116.7 (3)
C9—C14—H14A	109.4		

### Hydrogen-bond geometry (Å, °)

**Table:**

*D*—H···*A*	*D*—H	H···*A*	*D*···*A*	*D*—H···*A*
C13—H13B···O1^i^	0.96	2.39	3.344 (5)	172
C14—H14B···O4^ii^	0.96	2.52	3.472 (5)	174

Symmetry codes: (i) *x*+1, *y*−1, *z*; (ii) −*x*+1, −*y*, −*z*+1.
